# The Function and Photoregulatory Mechanisms of Cryptochromes From Moso Bamboo (*Phyllostachys edulis*)

**DOI:** 10.3389/fpls.2022.866057

**Published:** 2022-03-30

**Authors:** Ziyin Chen, Min Li, Siyuan Liu, Xiaojie Chen, Wenxiang Zhang, Qiang Zhu, Markus V. Kohnen, Qin Wang

**Affiliations:** ^1^College of Forestry, Basic Forestry and Proteomics Research Center, Fujian Agriculture and Forestry University, Fuzhou, China; ^2^College of Life Sciences, Fujian Agriculture and Forestry University, Fuzhou, China

**Keywords:** *Phyllostachys edulis*, light signaling, cryptochrome, PhePPK, PheBIC

## Abstract

Light is one of the most important environmental factors affecting growth and geographic distribution of forestry plants. Moso bamboo is the largest temperate bamboo on earth and an important non-woody forestry species that serves not only important functions in the economy of rural areas but also carbon sequestration in the world. Due to its decades-long reproductive timing, the germplasm of moso bamboo cannot be readily improved by conventional breeding methods, arguing for a greater need to study the gene function and regulatory mechanisms of this species. We systematically studied the photoregulatory mechanisms of the moso bamboo (*Phyllostachys edulis*) cryptochrome 1, PheCRY1. Our results show that, similar to its *Arabidopsis* counterpart, the bamboo PheCRY1s are functionally restricted to the blue light inhibition of cell elongation without an apparent activity in promoting floral initiation. We demonstrate that PheCRY1s undergo light-dependent oligomerization that is inhibited by PheBIC1s, and light-dependent phosphorylation that is catalyzed by PhePPKs. We hypothesize that light-induced phosphorylation of PheCRY1s facilitate their degradation, which control availability of the PheCRY1 proteins and photosensitivity of bamboo plants. Our results demonstrate the evolutionary conservation of not only the function but also photoregulatory mechanism of PheCRY1 in this monocot forestry species.

## Introduction

Bamboo is one of the most important non-timber forestry plants, covering 30 million hectares worldwide. Moso bamboo belongs to the grass family of Gramineae, but it is considered and often cultivated as an agroforestry and in recent years a carbon sequestering plant species. It has some remarkably unique characteristics, including the “fastest growing plant” record of any plant on the earth, among the tallest bamboo species, the long vegetative phase of more than 50 years before flowering, and the unique underground rhizome with a monopodial growth habit (Wilson and Loomis, 1967). There is a great need to improve bamboo agroforestry traits, but new bamboo cultivars cannot be readily generated through classical breeding methods, due to its long vegetative phase. A better understanding of the genes and gene functions of this species is critical to its future improvement.

Light is arguably the most critical environmental factor essential to both photosynthesis and plant development. Plants depend on blue light, red/far-red light, and UV-B photosensory receptor systems to control photosynthesis and development. Cryptochromes (CRY) are blue light receptors that control plant growth and development by mediating light-dependent regulation of gene expression (Wang and Lin, 2020). The prototype plant CRYs are *Arabidopsis* CRY1 and CRY2, which mediate primarily blue light inhibition of cell elongation and photoperiodic promotion of floral initiation (Ahmad and Cashmore, 1993; Mockler et al., 2003). It is previously reported that *Arabidopsis* CRY1 and CRY2 undergo these photoreactions: light-induced photo-oligomerization that is inhibited by the Blue-light Inhibitor of Cryptochromes (BICs), blue light-induced phosphorylation that is catalyzed by Photoregulatory Protein Kinases (PPKs), and polyubiquitination that is catalyzed by the E3 ubiquitin ligase Cul4^COP1/SPAs^ and Cul3^LRBs^ (Shalitin et al., 2002, 2003; Wang et al., 2016; Liu et al., 2017, 2020, 2022; Ma et al., 2020, 2021; Shao et al., 2020; Chen et al., 2021; Miao et al., 2021). Biological functions of CRYs have been reported in various plant species, including tomato (Ninu et al., 1999), soybean (Zhang et al., 2008; Lyu et al., 2021), Brassica (Chatterjee et al., 2006), pea (Platten et al., 2005), poplar (Mao et al., 2014), rice (Matsumoto et al., 2003), barley (Szucs et al., 2006), sorghum (Zhou et al., 2018), apple (Li et al., 2013a,b), and wheat (Xu et al., 2009). However, the photoregulatory mechanism of cryptochromes have not been studied in those species, and no photoreceptor has been previously reported in the monocot forestry species, such as bamboo. In the present study, we investigated different isoforms of moso bamboo (*Phyllostachys edulis*) PheCRY1. We show evolutionary conservation in not only function but also photoregulatory mechanisms of moso bamoo CRY1.

## Materials and Methods

### Accession Numbers

Moso bamboo gene sequences are obtained from the BambooGDB database^1^ under the accession numbers in Supplementary Table 1.

### Plasmid Construction

In-Fusion Cloning methods were used to generate the plasmids used in this study.^2^ The sequences subcloned into plasmids were verified by Sanger sequencing. The human cell expression vectors used in this study were pQCMV-Flag-GFP, pQCMV-GFP, and pCMV-Myc, which were described previously (Liu et al., 2017; Chen et al., 2021). pQCMV-YFP and pQCMV-mTagRFP-T vectors were modified from pQCMV-Flag-GFP by replacing GFP with YFP or mTagRFP-T (Supplementary Figure 1). To generate plasmids expressing *Flag-PheCRY1c*, *Flag-PheCRY1d*, and *Flag-PhePPKb*, the coding sequences (CDS) of *PheCRY1c* or *PheCRY1d* were amplified from moso bamboo cDNA, and the purified PCR products were then subcloned into *Spe*I/*Kpn*I-digested pQCMV-Flag-GFP vector through in-fusion. *Myc-PheCRY1c* and *Myc-PheCRY1d* plasmids were prepared by cloning the CDS of the genes into the *Bam*HI site of pCMV-Myc vector. For plasmids expressing *PheCRY1c-YFP* and *PheCRY1d-YFP*, the CDS of *PheCRY1c* and *PheCRY1d* were PCR-amplified from plasmids made before, and introduced into *Spe*I digested pQCMV-YFP vector by in-fusion. The *AtBIC1-mTagRFP* and *PheBIC1a-mTagRFP* plasmids were prepared by cloning the CDS of the *AtBIC1* or *PheBIC1a* genes into the *Spe*I-digested pQCMV-mTagRFP-T vector by in-fusion. The gene-specific primers for constructs used for expressing recombinant proteins in HEK293T cells are listed in Supplementary Table 2.

pFGFP binary vectors was used for creating overexpression transgenic lines (Chen et al., 2021). The pFGFP binary vector was modified from pCambia3301, which allows the interested genes to express under control of ACTIN 2 promoter and fuse with 2 × Flag and GFP tags. For generation of the plant binary plasmids of *FGFP-PheCRYs* and *FGFP-PheBIC1a*, the CDS of *PheCRY1s* and *PheBIC1a* were cloned into *Bam*HI-digested pFGFP vector. The primers for constructs used for plant transformation are included in Supplementary Table 2.

### Plant Materials and Growth Conditions

The wild-type *Arabidopsis* in this study is *rdr6-11*, which suppresses gene silencing (Peragine et al., 2004). The *cry1cry2rdr6* was described previously (Chen et al., 2021). *Arabidopsis* transgenic lines were generated *via Agrobacterium tumefaciens*-mediated the floral dip method (Clough, 2005). Plasmids of *FGFP-PheCRY1c* and *FGFP-PheCRY1d* were introduced into *cry1cry2rdr6* background to generate *FGFP-PheCRY1c* and *FGFP-PheCRY1d* overexpressing plants. *FGFP-BIC1a* overexpressing *Arabidopsis* was prepared in *rdr6-11* background. The transgenic lines were screened on nutrient soil with 25 mg/L Glufosinate-ammonium and lines with a relative high protein expression level were used for phenotype analysis.

**FIGURE 1:**
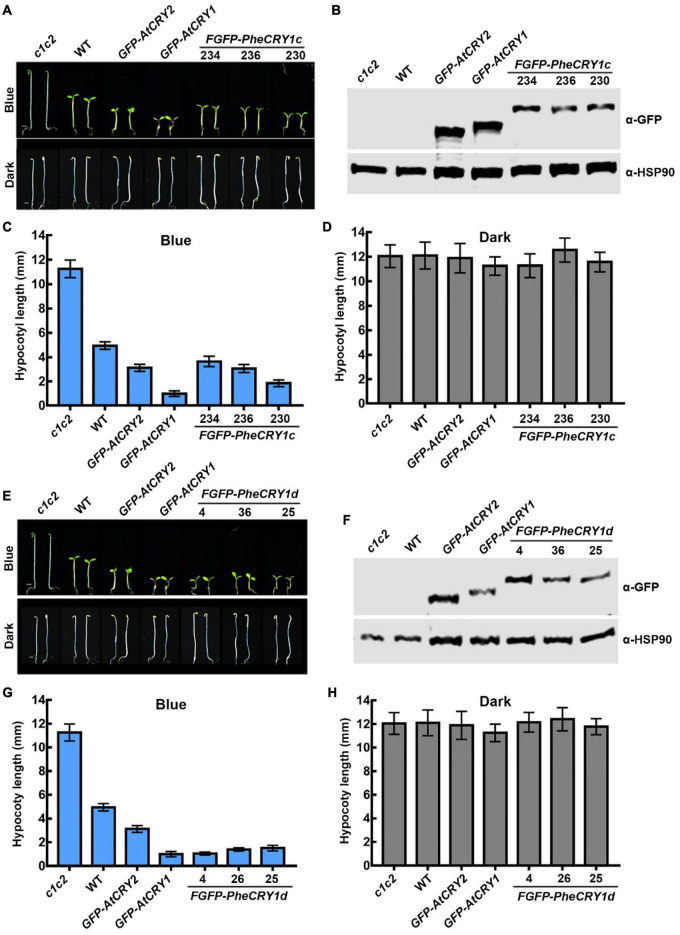
Bamboo PheCRY1s mediate blue light inhibition of hypocotyl elongation. **(A,E)** Images of 5-day-old *Arabidopsis* seedings grown under continuous blue light (10 μmolm^–2^s^–1^) or darkness. Scale bar, 1 mm. **(B,F)** Immunoblots showing the expression of FGFP-PheCRY1c **(B)** or FGFP-PheCRY1d **(F)** of seedlings shown in **(A)** or **(E)**. PheCRY1 and HSP90 were detected with anti-GFP antibody or anti-HSP90 antibody, respectively. HSP90 is used as a loading control. **(C,D)** Measurements of hypocotyl length of seedlings shown in **(A)**, (mean ± SD, *n* ≥ 20). **(G,H)** Measurements of hypocotyl length of seedlings shown in **(E)**, (mean ± SD, *n* ≥ 20).

For hypocotyl phenotype analysis, seedlings were grown on Murashige and Skoog medium (MS) plates with 1% sucrose at 20∼22°C for 5 days under different light conditions. The same representative images and quantification results of control seedlings, *cry1cry2*, wild-type (WT), *GFP-AtCRY1*, *GFP-AtCRY2*, were used for Figures 1, 5. For endogenous *CRY1* and *CRY2* degradation analysis in WT, *FGFP-PheBIC1a*, seedlings were grown in darkness on MS plates with 1% sucrose for 7 days, then subjected to 30 μmol m^–2^ s^–1^ blue light for the indicated time. For *FGFP-PheCRY1c* and *FGFP-PheCRY1d* and degradation analysis, seedlings were grown in darkness on MS plates with 1% sucrose for 6 days, then subjected to 100 μmol m^–2^ s^–1^ blue light for the indicated time. For flowering time measurements, seeds were sown in compound soil, stratified at 4°C in darkness for 3 days and then were transferred to long day (LD; 16 h light/8 h darkness) walk-in chambers.

### Protein Expression and Co-immunoprecipitation in HEK293T Cells

These methods were described previously (Chen et al., 2021).

### Photobody Formation Analysis

For photobody observation in HEK293T cells, cells were grown in 20 mm glass bottom confocal dishes (Cat. # 801001, NEST), and fixed in 4% formaldehyde for 15 min before analysis under confocal microscope. Hoechst 33342 (Cat. # 14533, Sigma-Aldrich) solution, at the concentration of 10 μg/ml, was used for nuclei staining. Leica TCS SP8 confocal microscope with a HC PL APO CS2 63x/1.40 OIL objective was used to captured the microscopic images. YFP signals were detected at 500–550 nm with 488 nm as excitation, mTagRFP was excited with 554 nm laser and detected at 566–629 nm. Hoechst 33342 was excited with 405 nm laser and detected at 420–480 nm.

### Blue Light-Induced Cryptochromes Degradation Assays

This method was described previously (Chen et al., 2021).

### Antibodies Used in This Study

The primary antibodies used in this study are as following: anti-AtCRY1 (1:5,000, homemade) (Lin et al., 1996), anti-AtCRY2 (1:3,000, homemade) (Lin et al., 1998), anti-HSP90 (1:20,000, cat # AbM51099-31-PU, Beijing Protein Innovation), anti-GFP (1:2,000, cat # 598, MBL), anti-Myc (1:2,000, cat # 05-724, Millipore), anti-Flag (1:1,500, cat # F3165, Sigma-Aldrich).

The secondary antibodies used in this study are as following: anti-Mouse-HRP (1:15,000, cat # 31430, Thermo Fisher Scientific), anti-Rabbit-HRP (1:15,000, cat # 31460, Thermo Fisher Scientific), goat anti-mouse Alexa Fluor 790 (1:15,000, cat # A11357), goat anti-rabbit IgG-Alexa Fluor 790 (1:15,000, cat # A11369), goat anti-mouse IgG-Alexa Fluor 680 (1:15,000, cat # A21058), goat anti-rabbit IgG-Alexa Fluor 680 (1:15,000, cat # A21109).

## Results

### Function of Moso Bamboo Cryptochromes

To investigate possible roles that cryptochromes play in moso bamboo, we searched moso bamboo (*Phyllostachys edulis*) genome sequence database, and identified five moso bamboo cryptochrome-like genes (*PheCRY*), *PheCRY1a*, *PheCRY1b*, *PheCRY1c*, *PheCRY1d*, and *PheCRY2* (Supplementary Figure 2A). PheCRY1a, PheCRY1b, PheCRY1c, and PheCRY1d show higher similarity to *Arabidopsis* cryptochrome 1 (AtCRY1, ∼ 72–84% identity) than that to *Arabidopsis* cryptochrome 2 (AtCRY2, ∼ 66–74% identity) (Supplementary Figure 2B). Similar to that found in cryptochromes of other plants, PheCRYs share more extensive sequence similarity in the N-terminal photolyase homologous chromophore-binding domains (PHR domain) than in the C-terminal domains (CCE domain) (Supplementary Figure 2C). We successfully cloned two representative *PheCRY* genes (*PheCRY1c* and *PheCRY1d*) for further biochemical and functional analysis.

Because the transformation technique has not been established for moso bamboo, we generated *FGFP-PheCRY1c* (with Flag and GFP tags fused to the N-terminus of PheCRY1c) and *FGFP-PheCRY1d* overexpression transgenic *Arabidopsis* in *cry1cry2* mutant background to test whether the bamboo CRY1s may complement the *Arabidopsis cry1cry2* mutant. Similar to previous studies of cryptochromes in other plants (Ninu et al., 1999; Matsumoto et al., 2003; Platten et al., 2005; Chatterjee et al., 2006; Szucs et al., 2006; Zhang et al., 2008; Xu et al., 2009; Li et al., 2013a,b; Mao et al., 2014; Zhou et al., 2018; Lyu et al., 2021), our study demonstrates that bamboo CRY1 serves similar function as the *Arabidopsis* CRYs in regulating cell elongation. Transgenic expression of *FGFP-PheCRY1c* and *FGFP-PheCRY1d* rescued the blue light-specific long hypocotyl phenotype of the *Arabidopsis cry1cry2* mutant, resulted in hypersensitivity to blue light as the *GFP-AtCRY1* and *GFP-AtCRY2* transgenic plants, which showed hypocotyls obviously shorter than that of the wild-type seedlings (Figure 1). Given that light inhibition of cell elongation is likely an ancient cellular response, it is not surprising that this activity of cryptochromes seems universally conserved in different plant species.

**FIGURE 2:**
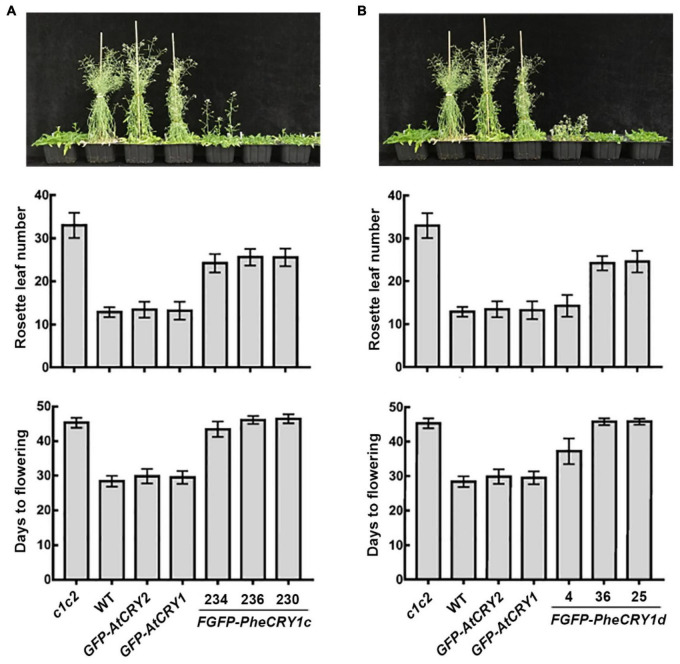
Bamboo PheCRY1 doesn’t function in the flowering time regulation in *Arabidopsis*. **(A,B)** Images of the flowering phenotypes of indicated plants grown in LD (16 h light/8 h dark) were shown. Rosette leaf numbers and days to flowering were recorded when flowering. Data were shown as mean ± SD, *n* ≥ 20.

However, bamboo CRY1 could not fully rescue the late-flowering phenotype of *Arabidopsis cry1cry2* double mutant (Figure 2). At the time of flowering, *FGFP-PheCRY1* transgenic *Arabidopsis* had less rosette leaves than that of *cry1cry2* mutant plants, but had more rosette leaves than that of wild-type and *GFP-AtCRY1* or *GFP-AtCRY2* overexpressing plants (Figure 2). Consistently, *FGFP-PheCRY1* transgenic *Arabidopsis* flowered at about the same time as the *cry1cry2* mutant (Figure 2). One transgenic line of *FGFP-PheCRY1d* (line #4) seemed to rescue the late-flowering phenotype of *Arabidopsis cry1cry2* mutant (Figure 2B), which is inconsistent with the flowering phenotype of other *FGFP-PheCRY1d* overexpressing lines and *FGFP-PheCRY1c* overexpressing lines. We reasoned that this inconsistence may cause by T-DNA insertion in the genomic region of a gene involving in flowering regulation, because all the transgenic lines of *FGFP-PheCRY1c* and *FGFP-PheCRY1d* showed similar physiological activity in hypocotyl inhibition. Since flowering time is a more recent evolutionary “invention” of angiosperm, the moso bamboo cryptochromes and *Arabidopsis* cryptochromes may have a distinct mode of action in the regulation of flowering time. It’s also possible that the flowering regulation mechanisms in moso bamboo are distinct from that of *Arabidopsis*, especially that bamboo has the extremely long vegetative phase and bamboo plants often take decades to flower (Numata, 1970; Lin et al., 2010). In *Arabidopsis*, CRY1 and CRY2 primarily mediate blue light inhibition of hypocotyl elongation and photoperiodic control of floral initiation, respectively (Ahmad and Cashmore, 1993; Mockler et al., 2003). It would also be interesting to investigate whether bamboo CRY2 may have the function in promoting floral initiation.

### Photoactivation of Moso Bamboo Cryptochromes

**FIGURE 3:**
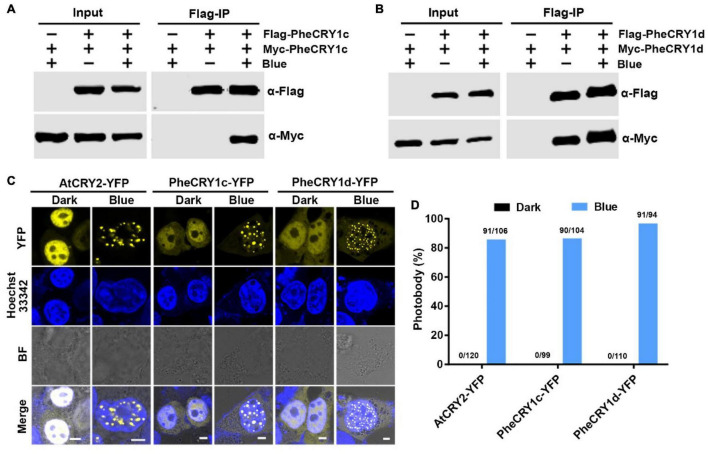
Bamboo PheCRY1s undergo blue light-enhanced homo-oligomerization. **(A,B)** Co-immunoprecipitation (co-IP) assays showing the blue light-enhanced homo-oligomerization of PheCRY1 in HEK293T cells. Cells co-expressing Flag-PheCRY1 and Myc-PheCRY1 were kept in the dark (Blue –) or exposed to 100 μmol^–2^s^–1^ of blue light for 2 h (Blue +). Immunoprecipitations (IP) were performed with Flag-conjugated beads. The IP and co-IP products were detected with anti-Flag and anti-Myc antibodies, respectively. **(C)** Confocal images showing the photobody formation of PheCRY1 in response to blue light in HEK293T cells. Cells were kept in the dark or exposed to blue light (100 μmol m^–2^ s^–1^) for 1 h. Before imaging, cells were fixed in 4% formaldehyde. Hoechst staining shows the nuclei. BF, bright field; scale bar, 5 μm. **(D)** Quantitative analysis of PheCRY1-YFP photobody formation in **(C)**. Photobody (%) is the percent of nuclei with photobodies among counted nuclei. The number of nuclei with photobodies and total counted nuclei was indicated above the columns.

Having demonstrated the blue light-dependent activity of PheCRY1c and PheCRY1d in transgenic *Arabidopsis* plants, we next asked whether the PheCRY1 may undergo similar photochemical reactions as that of *Arabidopsis* cryptochromes. Photo-oligomerization is the early photoreaction necessary for photoactivation of both CRY1 and CRY2 (Sang et al., 2005; Rosenfeldt et al., 2008; Wang et al., 2016; Liu et al., 2020). To investigate if the moso bamboo PheCRYs undergo homo-oligomerization in response to blue light, we examined the PheCRY1 oligomerization by co-expressing Flag-PheCRY1 and Myc-PheCRY1 in human embryonic kidney 293T (HEK293T) cells and tested the interaction of the two differentially tagged PheCRY1 by co-immunoprecipitation (co-IP) assay. In cells with similar amounts of Flag-PheCRY1 and Myc-PheCRY1 expression, Flag-beads coprecipitated more Myc-PheCRY1 in blue light than that in the dark (Figures 3A,B), demonstrating the blue light-enhanced PheCRY1 homo-oligomerization in the absence of other bamboo proteins. These results also demonstrated that, similar to *Arabidopsis* CRY1 and CRY2, bamboo PheCRY1 undergo oligomerization to become active. Photoexcited AtCRY1 and AtCRY2 are known to oligomerize into photobodies (Yu et al., 2009; Bugaj et al., 2013; Ozkan-Dagliyan et al., 2013; Wang et al., 2021; Liu et al., 2022), which is another early photoreaction of cryptochromes. We further investigated the PheCRY1 photobody formation in HEK293T cells. Figures 3C,D shows that PheCRY1-YFP (fused to yellow fluorescent protein) formed photobodies in the nucleus with blue light treatment, whereas no photobodies were detected in the cells in the dark, demonstrating that photoexcited PheCRY1 is capable of homo-oligomerization into photobodies in the absence of other proteins. These observations are also consistent with a hypothesis that photo-oligomerization might be an evolutionarily conserved photoactivation mechanism of cryptochrome photoreceptors. Taken together, our results demonstrate that moso bamboo PheCRY1 undergoes blue light-dependent homo-oligomerization to become photoactivated.

### Inactivation of Moso Bamboo Cryptochromes

**FIGURE 4:**
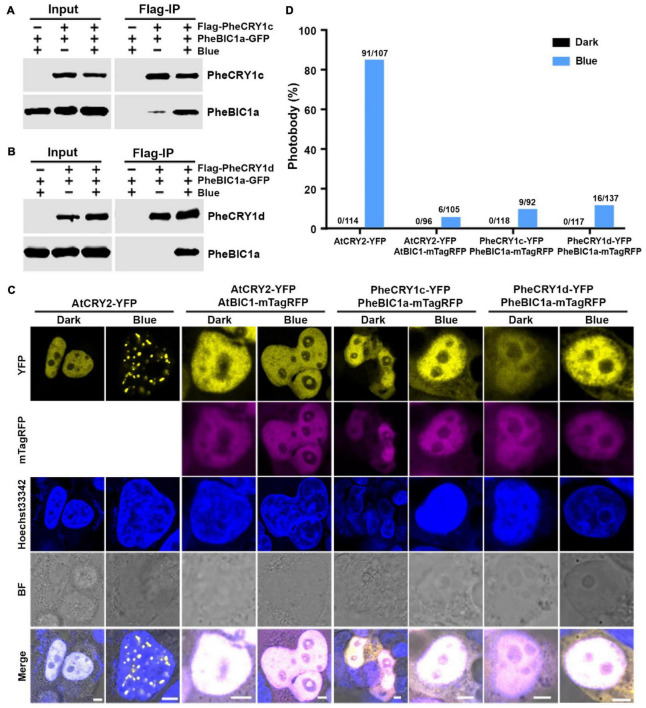
Bamboo PheBIC1 interacts with PheCRY1s to inhibit the photo-oligomerization of PheCRY1. **(A,B)** Co-immunoprecipitation (co-IP) assays showing the blue light-dependent interaction of PheCRY1 and PheBIC1 in HEK293T cells. Cells co-expressing Flag-PheCRY1 and PheBIC1-GFP were kept in the dark (Blue –) or exposed to 100 μmol^–2^s^–1^ of blue light for 2 h (Blue +). IP were performed with Flag-conjugated beads. The IP and co-IP products were detected with anti-Flag and anti-GFP antibodies, respectively. **(C)** Confocal images showing the inhibition of PheBIC1 on PheCRY1 photobody formation in HEK293T cells. Cells expressing indicated plasmid pairs were kept in the dark or exposed to blue light (100 μmol m^–2^ s^–1^) for 1 h. Cells were fixed in 4% formaldehyde before imaging. Hoechst staining shows the nuclei. BF, bright field; scale bar, 5 μm. **(D)** Quantitative analysis of the photobody formation in **(C)**. Photobody (%) is the percent of nuclei with photobodies among counted nuclei. The number of nuclei with photobodies and total counted nuclei was indicated above the columns.

Photoreceptor inactivation is an important photoregulatory mechanism for the maintenance of a sustained photosensitivity of the cells. *Arabidopsis* cryptochromes are inactivated by at least three mechanisms: BIC inhibition of oligomerization, dark-dependent monomerization, and blue light-dependent proteolysis (Yu et al., 2007; Wang et al., 2016; Liu et al., 2020, 2022; Chen et al., 2021). In *Arabidopsis*, the blue light-dependent cryptochrome oligomerization is suppressed by two closely related cryptochrome inhibitory proteins, BIC1 and BIC2. We examined whether there are BIC proteins in moso bamboo and whether the putative bamboo BIC proteins might suppress photo-oligomerization of the bamboo cryptochromes. Two highly homologous *PheBIC1* genes, *PheBIC1a* and *PheBIC1b*, were identified from the moso bamboo genome sequence database (Supplementary Figure 3). PheBIC1a and PheBIC1b share over 80% sequence identity and share more extensive sequence similarity in the CID domain (CRY-interacting domain) to *Arabidopsis* BIC1 and BIC2 (Supplementary Figure 3C), indicating that PheBICs may also function as cryptochrome inhibitors in moso bamboo. To test this hypothesis, we first examined the interaction of PheBIC1 and PheCRY1 in HEK293T cells co-expressing epitope-tagged PheBIC1 and PheCRY1 using co-IP assays. Our results show that blue light enhances PheBIC1 and PheCRY1 interaction. Both PheCRY1c and PheCRY1d coimmunoprecipitated PheBIC1a in HEK293T cells exposed to blue light, but little PheBIC1-PheCRY1 complex was coprecipitated in the dark (Figures 4A,B). This result suggests that PheBIC1 might interact with photoactivated PheCRY1 to inhibit their activities. We next tested this possibility by examining the effects of PheBIC1 on blue light-dependent PheCRY1 photobody formation by co-expressing PheBIC1-mTagRFP (fused to red fluorescent protein) and PheCRY1-YFP in HEK293T cells. AtCRY2-YFP and AtBIC1-mTagRFP co-expression cells were included as controls. Our results shows that no PheCRY1 photobodies were detected in the cells co-expressing PheBIC1 in blue light (Figures 4C,D), confirming our hypothesis that PheBIC1 act as cryptochrome-specific inhibitors in moso bamboo.

**FIGURE 5:**
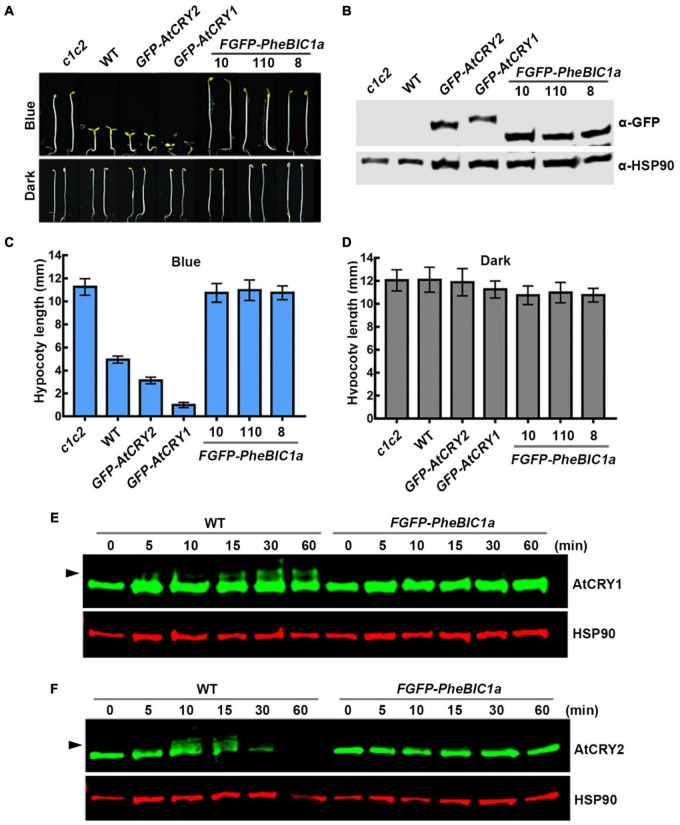
Bamboo PheBIC1 promotes hypocotyl elongation in blue light. **(A)** Images of 5-day-old *Arabidopsis* seedings grown under continuous blue light (10 μmolm^–2^s^–1^) or darkness. Scale bar, 1 mm. **(B)** Immunoblots showing the expression of FGFP-PheBIC1a of seedlings shown in **(A)**. PheBIC1 and HSP90 were detected with anti-GFP antibody or anti-HSP90 antibody, respectively. HSP90 is used as a loading control. **(C,D)** Measurements of hypocotyl length of seedlings shown in **(A)**, (mean ± SD, *n* ≥ 20). **(E,F)** Immunoblots showing the phosphorylation and degradation of endogenous *Arabidopsis* CRY1 and CRY2 in the wild-type or FGFP-PheBIC1a transgenic *Arabidopsis*. 7-day-old etiolated seedlings were irradiated with 30 μmolm^–2^ s^–1^ of blue light for the indicated time. Anti-AtCRY1, anti-AtCRY2 and anti-HSP90 antibodies were used to probe AtCRY1, AtCRY2 or HSP90. HSP90 is used as a loading control.

To test whether the bamboo BIC1 and their physical interaction with the bamboo CRY1 may be physiologically relevant, we prepared *FGFP-PheBIC1a* overexpression transgenic *Arabidopsis* in the wild-type background. The *PheBIC1a* overexpressing lines are insensitive to blue light, showing a long hypocotyl phenotype in blue light, resembling that of the *Arabidopsis cry1cry2* double mutant (Figures 5A–D). This result suggests that PheBIC1 may inhibit the function of *Arabidopsis* cryptochromes. We then investigated this hypothesis by comparing the phosphorylation and degradation of *Arabidopsis* endogenous CRY1 and CRY2 in the wild-type and *PheBIC1a* overexpressing plants. As expected, the blue light-dependent phosphorylation of both AtCRY1 and AtCRY2 were not detectable in *PheBIC1a* overexpressing *Arabidopsis* (Figures 5E,F), and the blue light-dependent degradation of AtCRY2 was also impaired in *PheBIC1a* overexpressing plants (Figure 5F). In the wild-type seedlings, obvious AtCRY1 phosphorylation was detected within 15 min of blue light exposure, while no AtCRY1 phosphorylation was detected even after 60 min of blue light exposure in the *PheBIC1a* overexpressing *Arabidopsis* (Figure 5E). The phosphorylation of AtCRY2 was detected within 10 min of blue light exposure and AtCRY2 protein was no detectable after 60 min of blue light exposure in the wild-type seedings (Figure 5F). However, neither phosphorylation nor degradation of AtCRY2 were detected in the *PheBIC1a* overexpressing *Arabidopsis* in response to blue light (Figure 5F). These results are consistent with our previously published results that overexpression of *Arabidopsis* BICs suppressed all known photobiochemical and photophysiological activities of AtCRY1 and AtCRY2, resulted in unrestrictive hypocotyl growth in blue light (Wang et al., 2016). Results of these experiments also indicate that PheBICs may be the PheCRYs-specific inhibitors to regulate the activity of PheCRYs in moso bamboo.

**FIGURE 6:**
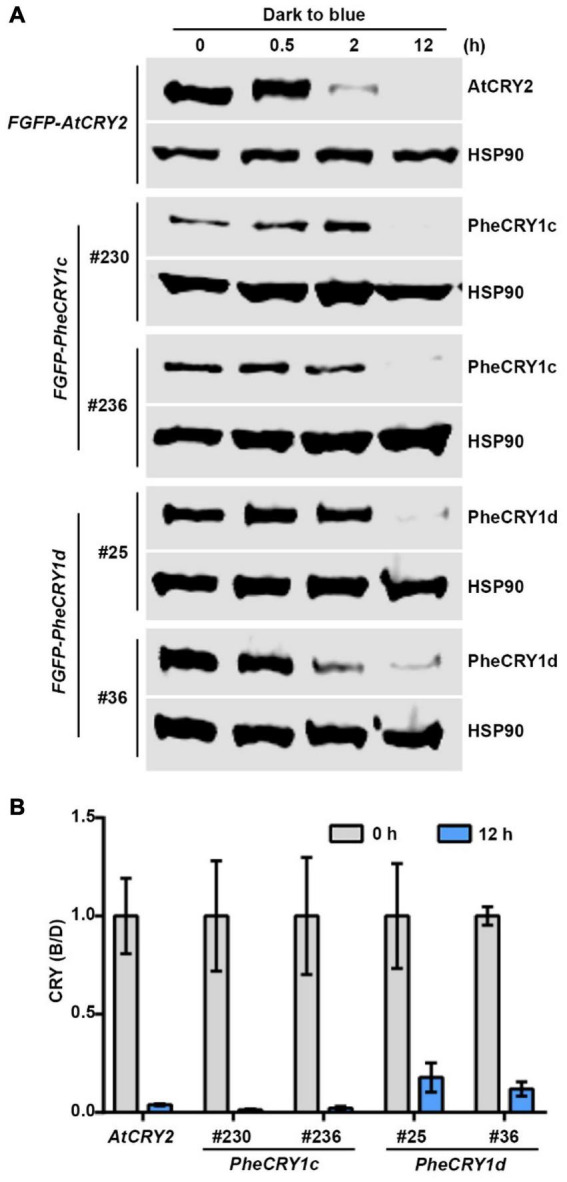
Bamboo PheCRY1s undergo blue light-dependent proteolysis. **(A)** Immunoblots showing the degradation of PheCRY1 in transgenic *Arabidopsis*. 6-day-old etiolated seedlings were irradiated with 100 μmolm^–2^ s^–1^ of blue light for the indicated time. Two independent transgenic lines were used for analysis. Anti-Flag antibody was used to detect AtCRY2 and PheCRY1. Anti-HSP90 antibody was used to detect HSP90. HSP90 is used as a loading control. **(B)** Quantitative analysis of CRY protein degradation at time 0 and 12 h of blue light treatment from immunoblots shown in **(A)**. CRY (B/D) = [CRY/HSP90]^blue^/[CRY/HSP90]^dark^. Data are presented as mean ± SD (*n* = 3 individual immunoblots).

Blue light-dependent proteolysis serve as another inactivation mechanism of crytochromes by removing the activated cryptochromes in cells. Depending on the plant species, CRY1, CRY2, and other types of cryptochromes have all been shown to undergo light-dependent proteolysis (Lin et al., 1998; Imaizumi et al., 2000; Reisdorph and Small, 2004; Chatterjee et al., 2006; Hirose et al., 2006). In *Arabidopsis*, both AtCRY1 and AtCRY2 undergo blue light-dependent degradation in the nucleus (Yu et al., 2007; Liu et al., 2022). We next examined the protein degradation of PheCRY1 in transgenic *Arabidopsis*. Figure 6 showed that in etiolated seedlings exposed to blue light, the PheCRY1c and PheCRY1d protein exhibits a pronounced decrease after 12 h of blue light treatment. The rate of blue light-induced degradation of PheCRY1c and PheCRY1d were slower than that of the AtCRY2, which showed apparent degradation after 2 h of blue light treatment. We reasoned that PheCRY1c and PheCRY1d might be more closely related to *Arabidopsis* CRY1, which requires higher intensities of blue light and longer blue light treatment time to induce its degradation. Our results also suggest that the light-dependent proteolysis may be another evolutionary conserved mechanism for inactivation of PheCRYs in moso bamboo, and the E3 ubiquitin ligases, CUL3^LRBs^ and CUL4^COP1/SPAs^, responsible for *Arabidopsis* cryptochromes degradation may also be conserved between *Arabidopsis* and moso bamboo. However, this hypothesis remains to be tested.

### Phosphorylation of Moso Bamboo Cryptochromes

**FIGURE 7:**
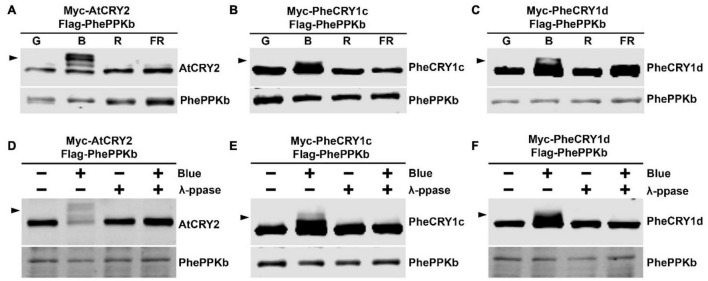
Bamboo PhePPKb catalyzes the blue light-dependent phosphorylation of bamboo PheCRY1 in HEK293T cells. **(A–C)** Cells co-expressing indicated plasmid pairs were exposed to green light (G, 50 μmolm^–2^ s^–1^), blue light (B, 50 μmolm^–2^ s^–1^), red light (R, 50 μmolm^–2^ s^–1^), or far-red light (FR, 5 μmolm^–2^ s^–1^) for 60 min. Immunoblots were probed with the anti-Myc or anti-Flag antibodies. **(D–F)** Cells co-expressing indicated plasmids were kept in the dark (Blue –) or treated with 100 μmolm^–2^ s^–1^ of blue light for 2 h (Blue +). Lysates were treated without (- λ-PPase) or with λ-PPase (+ λ-PPase), and analyzed by immunoblots probed with the anti-Flag or anti-Myc antibodies. Arrowheads indicate the phosphorylated CRY.

Photoexcited *Arabidopsis* CRY1 and CRY2 further undergo blue light-dependent phosphorylation to both enhance their activities and facilitate their ubiquitination and degradation in the nucleus (Shalitin et al., 2002, 2003; Yu et al., 2007; Weidler et al., 2012; Liu et al., 2016, 2022; Miao et al., 2021). Four closely related plant-specific PPKs (PPK1, PPK2, PPK3, and PPK4) have been shown to catalyze the phosphorylation of *Arabidopsis* CRY1 and CRY2 in blue light (Liu et al., 2017; Ni et al., 2017). To test whether moso bamboo PheCRYs undergo blue light-induced phosphorylation and whether PhePPKs are the protein kinases catalyzing light-induced phosphorylation of PheCRYs, we searched moso bamboo genome sequence database, and found 10 closely related *PhePPKs* (*PhePPKa*–*PhePPKj*) genes in moso bamboo (Supplementary Figure 4). Similar to *Arabidopsis* PPKs, PhePPKs have two domains, a N-terminal Ser/Thr kinase domain which is homolog to casein kinase 1 (CK1) (Liu et al., 2017), and a C-terminal domain that are highly conserved within the PPK gene families (Supplementary Figure 4C). We cloned PhePPKb and examine its catalytic characteristics in HEK293T cells. We co-expressed *Arabidopsis* CRY2 or moso bamboo PheCRY1 with AtPPK1 or PhePPKb in HEK293T cells to investigate the catalytic activity of PPK on CRY by electrophoretic mobility upshift immunoblot assays (Shalitin et al., 2002, 2003; Wang et al., 2016). AtCRY2, PheCRY1c, and PheCRY1d exhibit retarded migration in cells co-expressing PhePPKb or AtPPK1 treated with blue light, whereas no migration upshift was detected in cells treated with green light, red light or far-red light (Figures 7A–C and Supplementary Figures 5A–C). The blue light-specific upshift migration of CRYs disappeared after protein phosphatase treatments (Figures 7D–F and Supplementary Figures 5D–F), confirming the slow migration bands were phosphorylated PheCRY1. We also found that AtCRY2 can be phosphorylated by moso bamboo PhePPK1, and AtPPK1 can phosphorylate bamboo PheCRY1 (Figures 7A,D and Supplementary Figures 5B,C,E,F), indicating that PPK-CRY (enzyme-substrate) pairing is more likely determined by structure-based mechanisms regardless of plant species. In summary, our results demonstrate that moso bamboo CRY1s undergo blue-light dependent phosphorylation *in vitro*, and PhePPKs are the protein kinases that catalyze the blue-light dependent phosphorylation of moso bamboo cryptochromes, indicating that the molecular basis for light-dependent phosphorylation of cryptochrome is also evolutionary conserved between *Arabidopsis* and moso bamboo.

## Discussion

Although the function of CRYs have been reported in many plant species, the photoregulatory mechanisms of CRYs have been study for primarily the *Arabidopsis* CRYs. A major goal of our present study is to address the question whether the photoregulatory mechanism of plant CRYs are evolutionarily conserved. Our results demonstrate that at least three photoregulatory mechanisms of bamboo CRY1 are evolutionarily conserved. First, bamboo CRY1 undergo blue light-dependent homo-oligomerization as that of *Arabidopsis* CRY2, and this photoreaction is inhibited by the negative regulator of bamboo BICs. Second, bamboo CRY1 also undergoes blue light-induced phosphorylation that can be catalyzed by the bamboo PPK kinases. Third, bamboo CRY1 proteins undergo light-induced proteolysis as that of the *Arabidopsis* CRYs. Our results that bamboo CRY1 are phosphorylated and degraded in *Arabidopsis* and that bamboo PPKb catalyze light-induced phosphorylation of *Arabidopsis* CRY1 demonstrate the evolutionary conservation of this photoregulatory mechanism. Based on these results, we hypothesize that the photoregulatory mechanism of plant CRYs are predominantly conserved during evolution.

## Data Availability Statement

The raw data supporting the conclusions of this article will be made available by the authors, without undue reservation.

## Author Contributions

ZC performed most of the experiments and wrote part of the manuscript. ML generated genetic materials and collected the phenotypic data. SL helped with photobody formation analysis. XC and WZ repeated some results. QZ helped with bamboo transformation. MK provided financial supports. QW conceived the project, designed the experiments, and wrote the manuscript. All authors contributed to the article and approved the submitted version.

## Conflict of Interest

The authors declare that the research was conducted in the absence of any commercial or financial relationships that could be construed as a potential conflict of interest.

## Publisher’s Note

All claims expressed in this article are solely those of the authors and do not necessarily represent those of their affiliated organizations, or those of the publisher, the editors and the reviewers. Any product that may be evaluated in this article, or claim that may be made by its manufacturer, is not guaranteed or endorsed by the publisher.

## Footnotes

1
www.bamboogdb.org

2
https://www.takarabio.com/products/cloning/in-fusion-cloning
