# Supplementary material for: The Function and Photoregulatory Mechanisms of Cryptochromes From Moso Bamboo (Phyllostachys edulis)
Source: Front Plant Sci. 2022 Mar 30;13:866057. doi: 10.3389/fpls.2022.866057 (PMC9006058; doi:10.3389/fpls.2022.866057)
Supplement: Supplementary file 1 [file Data_Sheet_1.PDF]

Supplementary Material

Supplementary Figure 1. Maps of vectors used in this study.

Supplementary Figure 2. Homology analysis of cryptochromes from Arabidopsis and moso bamboo.

Supplementary Figure 3. Homology analysis of BICs from Arabidopsis and moso bamboo.

Supplementary Figure 4. Homology analysis of PPKs from Arabidopsis and moso bamboo.

Supplementary Figure 5. Arabidopsis AtPPK1 catalyzes the blue light-dependent phosphorylation of bamboo PheCRY1 in HEK293T cells.

Supplementary Table 1. Accessions of genes in this study.

Supplementary Table 2. List of primers used in this study.

Supplementary Figure 1

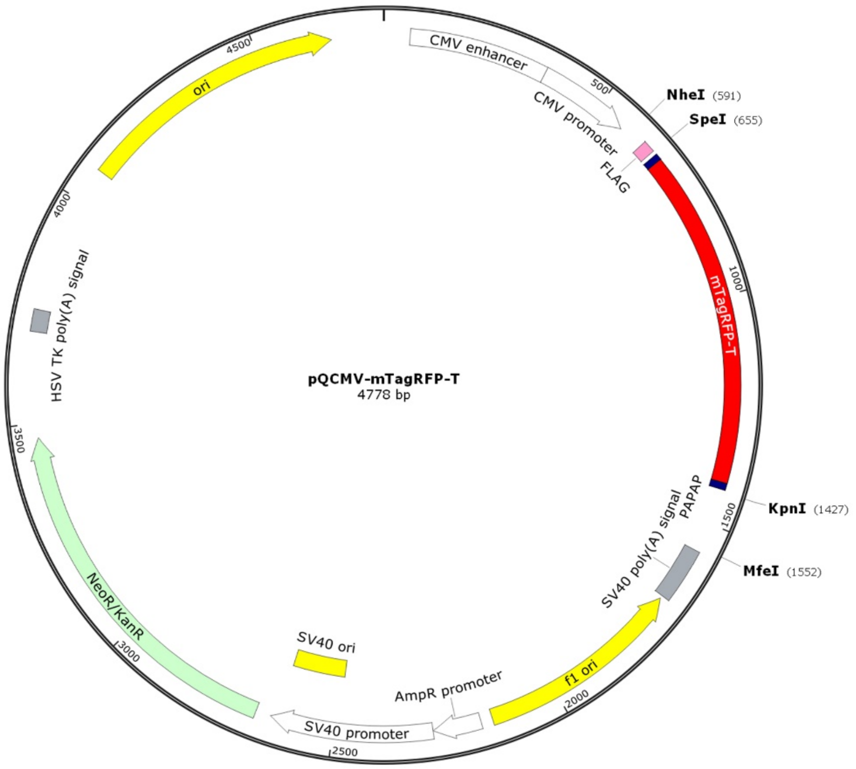

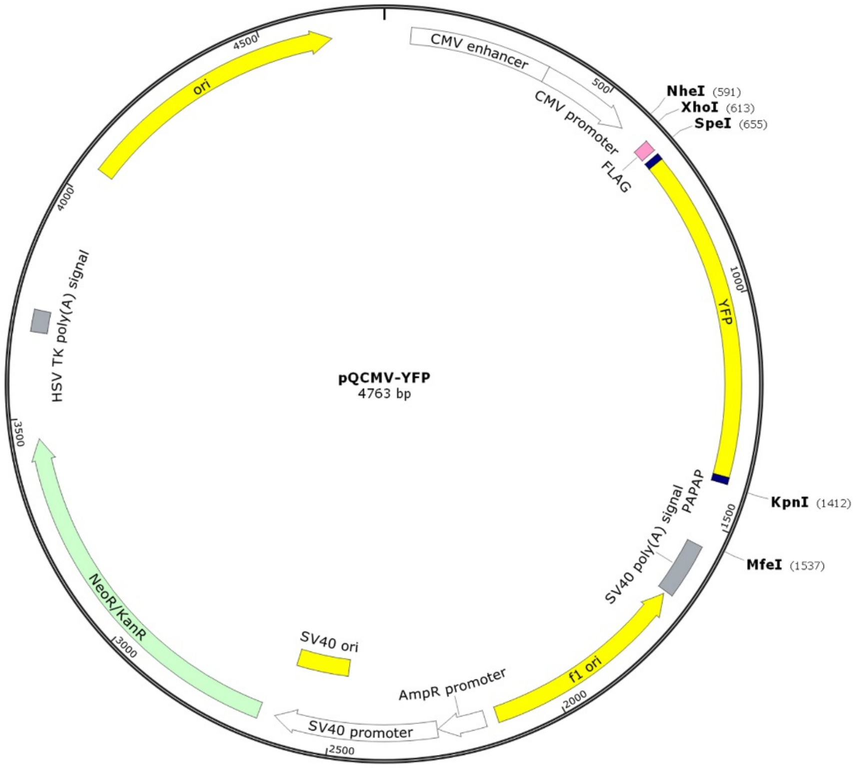

Supplementary Figure 1. Maps of vectors used in this study.

Supplementary Figure 2

A

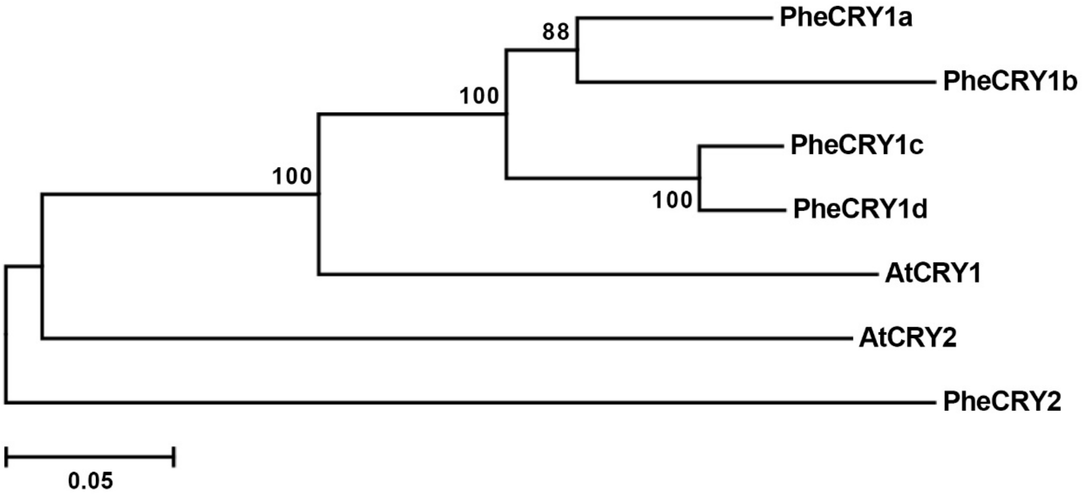

B

Percent Identity (upper right); Divergence (lower left)

	1	2	3	4	5	6	7		
1		74.1	79.0	72.0	83.5	83.3	13.9	1	AtCRY1
2	31.8		71.4	66.4	73.8	73.8	14.2	2	AtCRY2
3	24.7	35.9		85.9	87.7	87.2	19.4	3	PheCRY1a
4	35.0	44.4	15.7		78.3	77.8	26.5	4	PheCRY1b
5	18.7	32.3	13.5	25.6		97.3	14.6	5	PheCRY1c
6	18.9	32.3	14.0	26.4	2.7		14.9	6	PheCRY1d
7	354.0	347.0	254.0	185.2	337.0	332.0		7	PheCRY2
	1	2	3	4	5	6	7		

Supplementary Figure 2 – continued

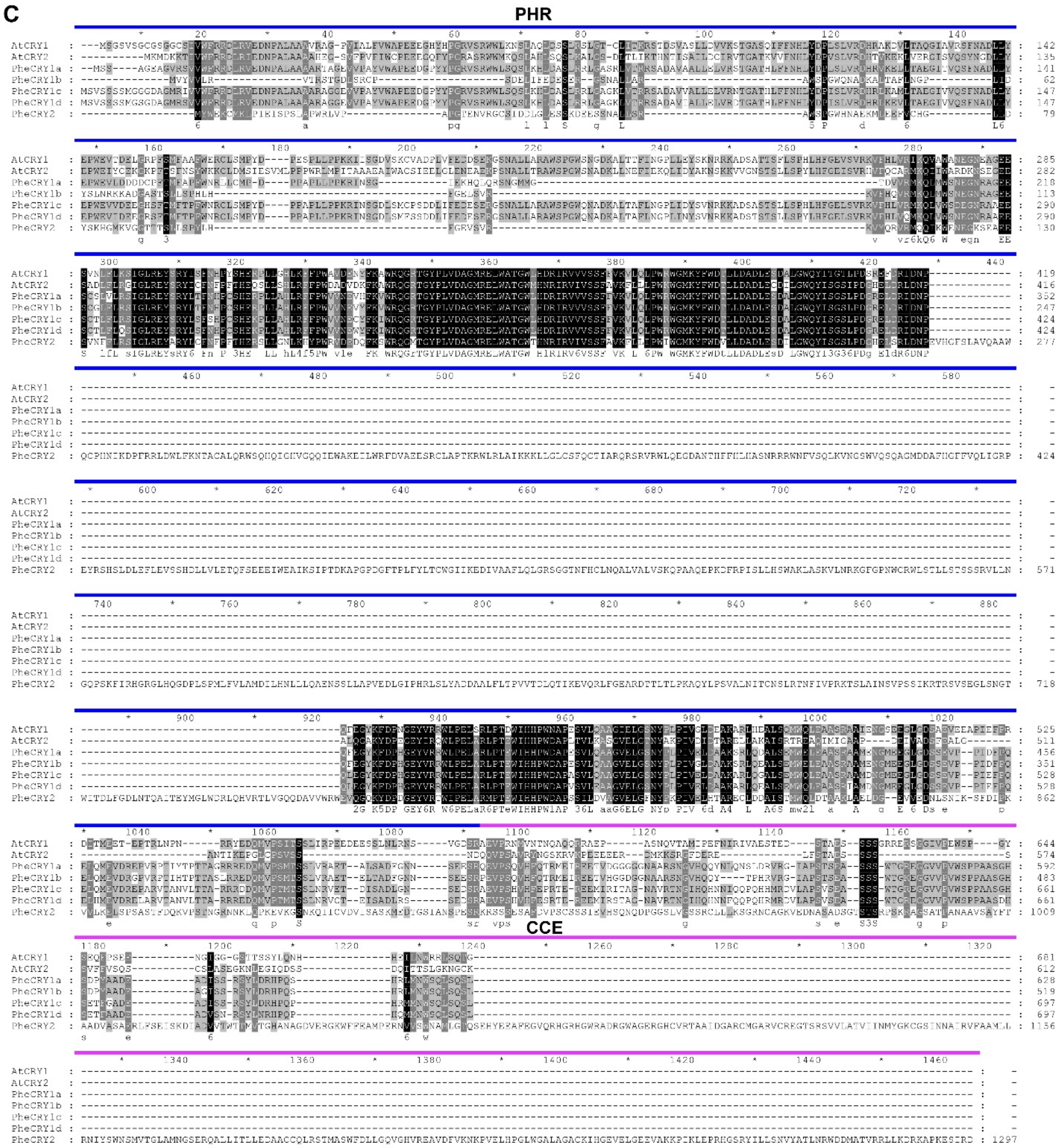

Supplementary Figure 2. Homology analysis of cryptochromes from Arabidopsis and moso bamboo.

(A) An un-rooted phylogenetic tree was generated by the neighbor-joining method using the amino acid sequences of cryptochromes from Arabidopsis and moso bamboo (*Phyllostachys edulis*).

(B) The percent identity and divergence between cryptochromes from Arabidopsis and moso bamboo, analyzed by MegAlign.

(C) The full-length amino acid sequences alignment of cryptochromes from Arabidopsis and moso bamboo. PHR, photolyase homologous region; CCE, CRY C-terminal extension.

Supplementary Figure 3

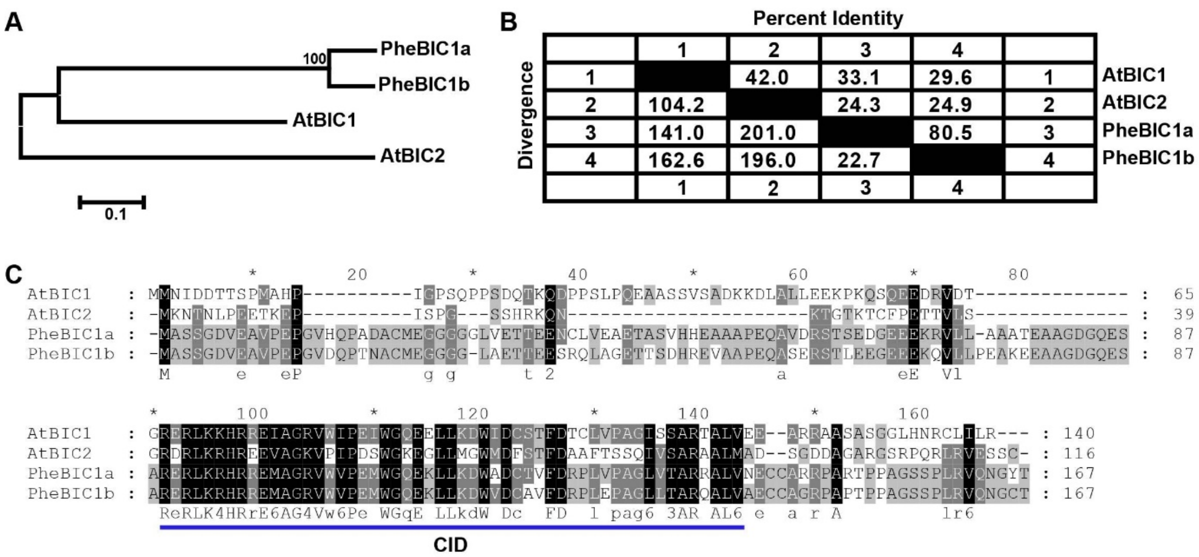

Supplementary Figure 3. Homology analysis of BICs from Arabidopsis and moso bamboo.

(A) An un-rooted phylogenetic tree was generated by the neighbor-joining method using the amino acid sequences of BICs from Arabidopsis and moso bamboo (*Phyllostachys edulis*).

(B) The percent identity and divergence between BICs from Arabidopsis and moso bamboo, analyzed by MegAlign.

(C) The full-length amino acid sequences alignment of BICs from Arabidopsis and moso bamboo. CID, cryptochrome interacting domain.

Supplementary Figure 4

A

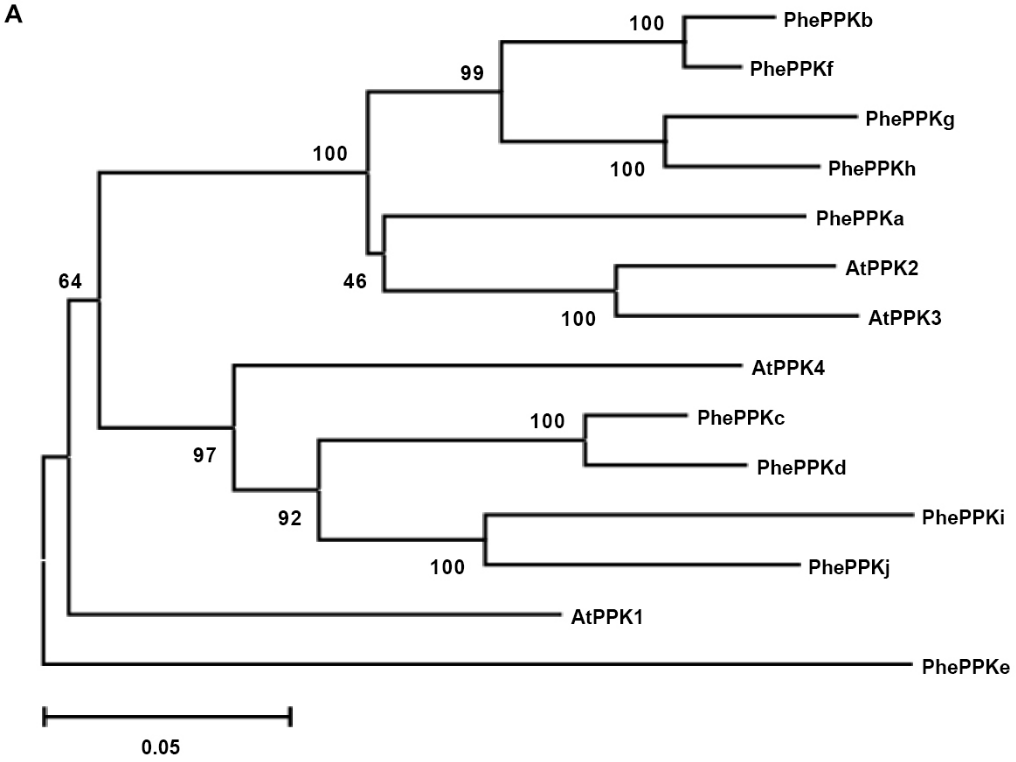

B

Percent Identity (upper triangle) / Divergence (lower triangle)

	1	2	3	4	5	6	7	8	9	10	11	12	13	14		
1		74.0	74.2	74.6	67.5	69.0	71.3	73.5	66.7	71.7	45.5	56.1	69.8	71.0	1	AtPPK1
2	32.0		91.3	70.4	75.4	76.3	68.5	71.3	63.0	78.3	49.2	60.6	68.4	70.9	2	AtPPK2
3	31.7	9.2		71.0	76.5	76.6	69.0	71.3	62.1	78.7	49.2	60.3	68.5	70.3	3	AtPPK3
4	31.0	37.5	36.7		65.3	66.1	76.1	79.1	65.0	68.4	42.7	54.7	74.5	76.8	4	AtPPK4
5	42.6	29.9	28.3	46.4		75.7	66.1	65.0	55.5	74.2	51.0	55.0	61.9	63.7	5	PhePPKa
6	40.0	28.6	28.1	45.0	29.4		66.3	67.1	57.7	89.9	56.3	60.7	62.3	65.1	6	PhePPKb
7	36.1	40.7	40.1	28.8	45.0	44.6		89.3	62.0	68.4	45.5	52.9	74.8	77.0	7	PhePPKc
8	32.8	36.2	36.2	24.5	46.9	43.1	11.6		65.0	72.3	45.1	57.6	78.5	80.2	8	PhePPKd
9	43.8	50.7	52.4	46.9	66.4	61.4	52.5	46.9		60.2	35.1	45.9	63.7	66.0	9	PhePPKe
10	35.5	25.7	25.1	40.9	31.6	10.8	40.9	34.6	56.2		55.7	66.6	68.3	71.0	10	PhePPKf
11	92.7	82.0	82.0	101.7	77.2	64.6	92.7	94.1	132.1	65.8		61.6	41.0	43.9	11	PhePPKg
12	64.9	55.3	55.9	68.1	67.5	55.0	72.5	61.6	91.4	44.0	53.3		54.1	56.9	12	PhePPKh
13	38.5	40.9	40.7	31.2	52.8	52.0	30.8	25.4	49.2	41.1	107.7	69.6		85.5	13	PhePPKi
14	36.6	37.7	37.7	27.9	49.3	46.7	27.5	23.0	45.1	36.7	97.8	63.2	16.2		14	PhePPKj
	1	2	3	4	5	6	7	8	9	10	11	12	13	14		

Supplementary Figure 4 – continued

C

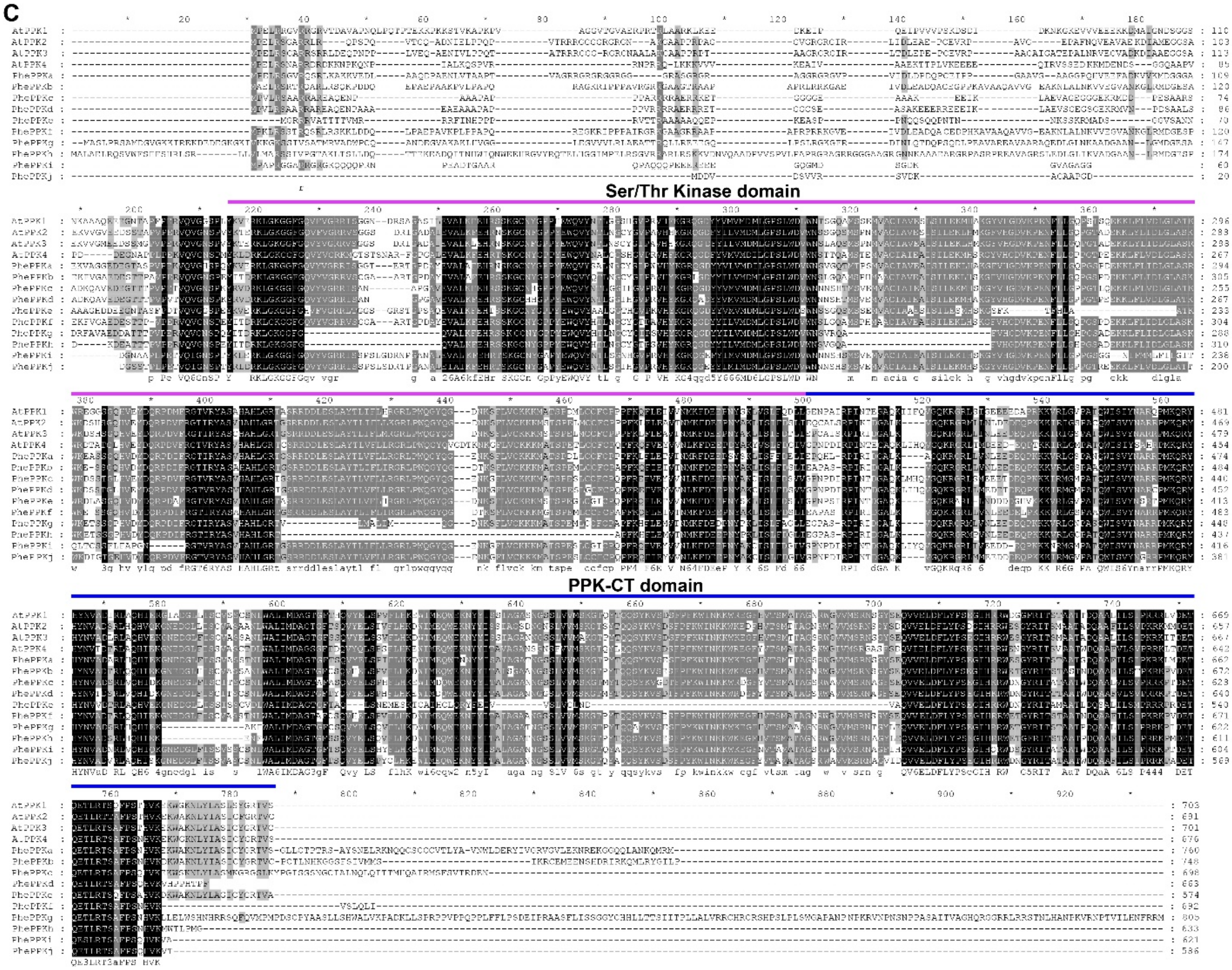

Supplementary Figure 4. Homology analysis of PPKs from Arabidopsis and moso bamboo.

(A) An un-rooted phylogenetic tree was generated by the neighbor-joining method using the amino acid sequences of PPKs from Arabidopsis and moso bamboo (*Phyllostachys edulis*).

(B) The percent identity and divergence between PPKs from Arabidopsis and moso bamboo, analyzed by MegAlign.

(C) The full-length amino acid sequences alignment of PPKs from Arabidopsis and moso bamboo.

Supplementary Figure 5

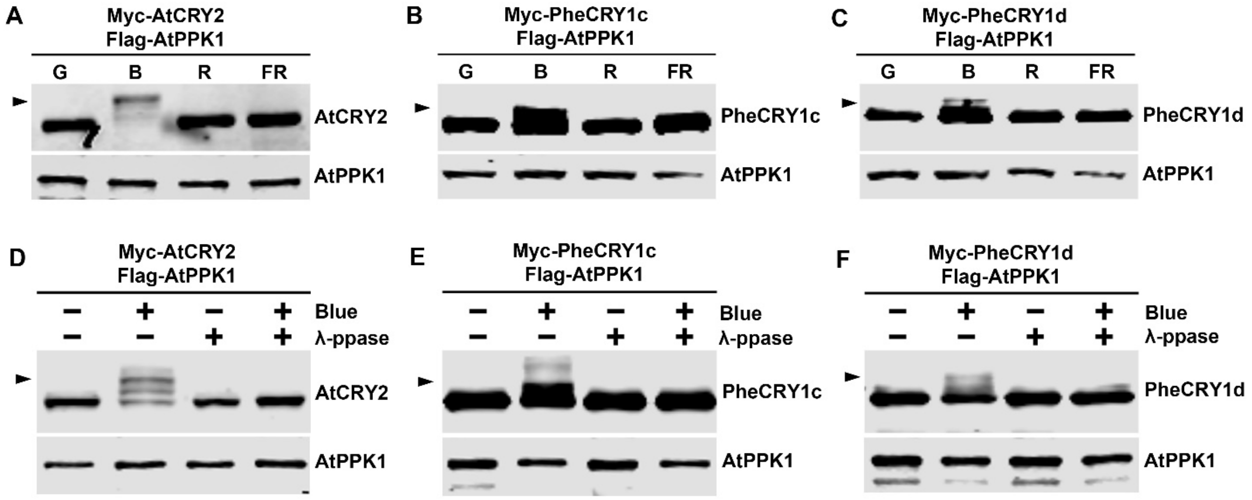

Supplementary Figure 5. Arabidopsis AtPPK1 catalyzes the blue light-dependent phosphorylation of bamboo PheCRY1 in HEK293T cells.

(A-C) Cells co-expressing indicated plasmid pairs were exposed to green (G, 50 µmolm^{-2} s^{-1}), blue (B, 50 µmolm^{-2} s^{-1}), red (R, 50 µmolm^{-2} s^{-1}) or far-red light (FR, 5 µmolm^{-2} s^{-1}) for 60 min. Immunoblots were probed with the anti-Myc or anti-Flag antibodies.

(D-E) Cells co-expressing indicated plasmids were kept in the dark (Blue -) or treated with 100 µmolm^{-2} s^{-1} of blue light for 2 hours (Blue +). Lysates were treated without (- λ-PPase) or with λ-PPase (+ λ-PPase), and analyzed by immunoblots probed with the anti-Flag or anti-Myc antibodies. Arrowheads indicate the phosphorylated CRY.

Supplementary Table 1. Accessions of genes in this study.

Gene	Accession	CDS Size(bp)
AtCRY1	AT4G08920.1	2,046
AtCRY2	AT1G04400.2	1,839
AtBIC1	AT3G44450.1	351
AtBIC2	AT3G44450.1	351
AtPPK1	AT3G13670.1	2,112
AtPPK2	AT5G18190.1	2,076
AtPPK3	AT3G03940.1	2,106
AtPPK4	AT2G25760.2	2,031
PheCRY1a	PH01000263G1210	1,887
PheCRY1b	PH01002373G0140	1,560
PheCRY1c	PH01000349G1020	2,094
PheCRY1d	PH01000968G0540	2,094
PheCRY2	PH01002304G0120	3,894
PheBIC1a	PH01000265G0010	504
PheBIC1b	PH01000144G0360	504
PhePPKa	PH01000775G0130	2,283
PhePPKb	PH01001103G0290	2,247
PhePPKc	PH01000284G0460	2,097
PhePPKd	PH01000252G1050	1,992
PhePPKe	PH01000134G1480	1,725
PhePPKf	PH01001702G0280	2,079
PhePPKg	PH01000174G1150	2,418
PhePPKh	PH01001405G0250	1,902
PhePPKi	PH01003150G0120	1,866
PhePPKj	PH01000045G1690	1,761

Supplementary Table 2. List of primers used in this study.

Primer Name	Primer Sequence (5'-3')
PheCRY1c-YFP F	CACCATGGCTACTAGTATGTCGGTCTCGTCCTCGTC
PheCRY1c-YFP R	CTGGAGCTGGACTAGTCAATGACTGTGATAGCTGACTCCA
PheCRY1d-YFP F	CACCATGGCTACTAGTATGTCGGTCTCGTCCTCGTC
PheCRY1d-YFP R	CTGGAGCTGGACTAGTCAATGACTGTGATAGCTGACTCCA
pQCMV PheBIC1a-mtagRFP F	CACCATGGCTACTAGTATGGCGTCCTCCGGCGACGT
pQCMV PheBIC1a-mtagRFP R	CTGGAGCTGGACTAGTGGTGTAGCCGTTTTGCACCC
pQCMV AtBIC1-mtagRFP F	CACCATGGCTACTAGTATGATGAACATCGACGATACG
pQCMV AtBIC1-mtagRFP R	CTGGAGCTGGACTAGTACGTAAGATCAAGCAACGATTATG
pQCMV PhBIC1a-GFP F	AGCTCCAGCTGGTACCATGTGCATTAGCAGGAAGCAAGT
pQCMV PheBIC1a-GFP R	CGTGCTCAGCGGTACCTTTATTCTAAAGCTACCTGTATGATTTTGAGCT
pFGFP-PheCRY1b F	TCCAGCTCCAGGATCCATGGTGTATGTTGTGCTCCGT
pFGFP-PheCRY1b R	GAGAAAGCTTGGATCCTCACAATGACTGCGAGAGTTGACT
pFGFP-PheCRY1c F	TCCAGCTCCAGGATCCATGTCGGTCTCGTCCTCGT
pFGFP-PheCRY1c R	GAGAAAGCTTGGATCCTCACAATGACTGTGATAGCTGACTCCA
pFGFP-PheCRY1d F	TCCAGCTCCAGGATCCATGTCGGTCTCGTCCTCGT
pFGFP-PheCRY1d R	GAGAAAGCTTGGATCCTCACAATGACTGTGATAGCTGACT
pFGFP-PheCRY3 F	TCCAGCTCCAGGATCCATGAGCGCTGCCTCGAGCTCGA
pFGFP-PheCRY3 R	GAGAAAGCTTGGATCCCTACTTTTGTCTTATGTACATG
pQCMVFlag-PheCRY1b F	CGACAAGGCTACTAGTATGGTGTATGTTGTGCTCCGT
pQCMVFlag-PheCRY1b R	CGTGCTCAGCGGTACCTCACAATGACTGCGAGAGTTGACT
pQCMVFlag-PheCRY1c F	CGACAAGGCTACTAGTATGTCGGTCTCGTCCTCGT
pQCMVFlag-PheCRY1c R	CGTGCTCAGCGGTACCTCACAATGACTGTGATAGCTGACTCCA
pQCMVFlag-PheCRY1d F	CGACAAGGCTACTAGTATGTCGGTCTCGTCCTCGT
pQCMVFlag-PheCRY1d R	CGTGCTCAGCGGTACCTCACAATGACTGTGATAGCTGACT
pQCMVFlag-PhePPKb F	CGACAAGGCTACTAGTATGGCAGAGCTGCGAAGCAGAA
pQCMVFlag-PhePPKb R	CGTGCTCAGCGGTACCTTACGGTAGGATACCATACCGCAAC
pCMVMyc-PheCRY1c F	GGAGGACCTGGGATCCATGTCGGTCTCGTCCTCGT
pCMVMyc-PheCRY1c R	TAGCAGGCCTGGATCCTCACAATGACTGTGATAGCTGACTCCA
pCMVMyc-PheCRY1d F	GGAGGACCTGGGATCCATGTCGGTCTCGTCCTCGT
pCMVMyc-PheCRY1d R	TAGCAGGCCTGGATCCTCACAATGACTGTGATAGCTGACT
