# Supplementary figures and images for: The Function and Photoregulatory Mechanisms of Cryptochromes From Moso Bamboo (Phyllostachys edulis)
Source: Front Plant Sci. 2022 Mar 30;13:866057. doi: 10.3389/fpls.2022.866057 (PMC9006058; doi:10.3389/fpls.2022.866057)

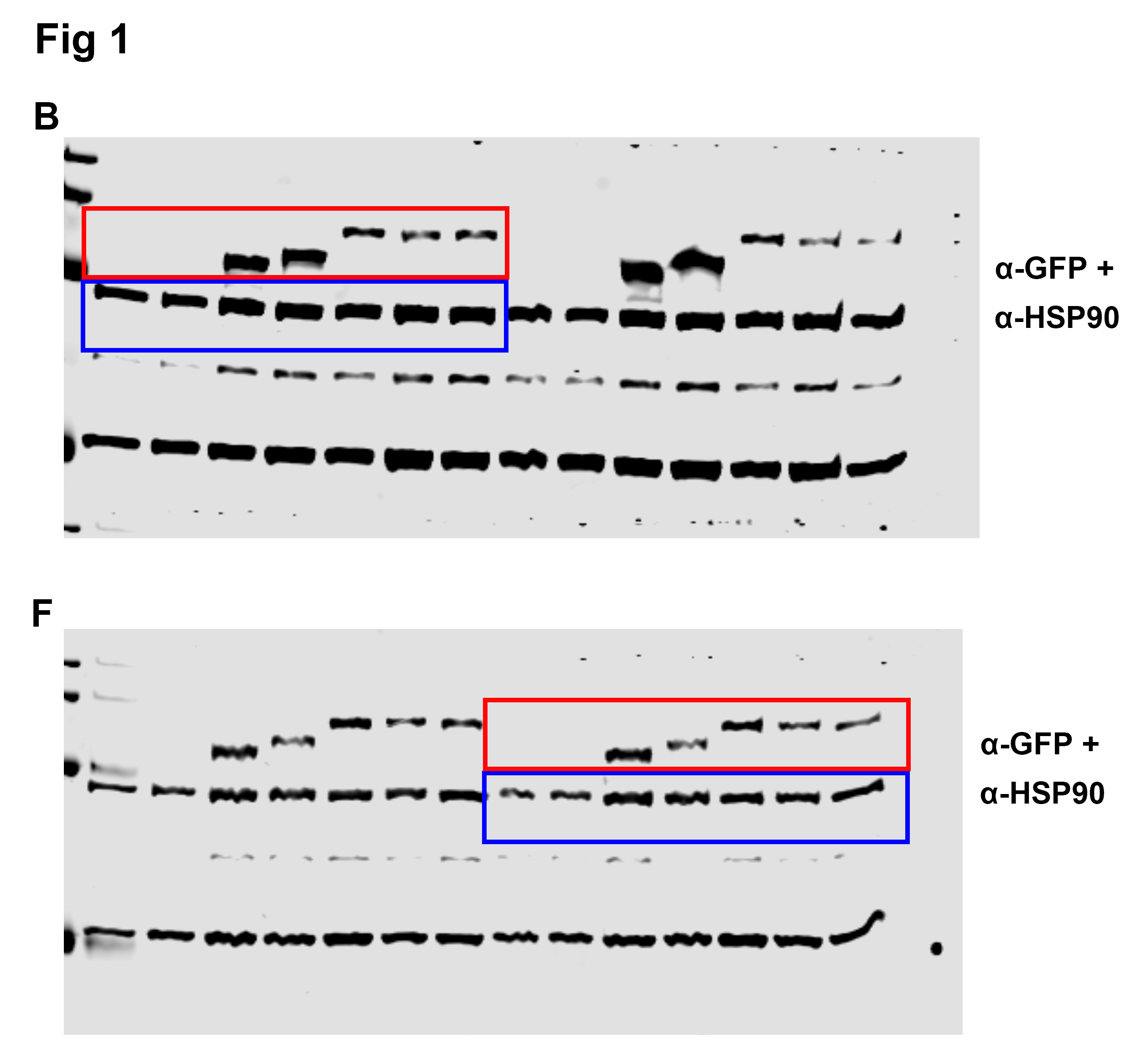

Supplement: Supplementary file 2 [file Data_Sheet_2.ZIP › Original WB images for 866057-2/Figure 1 original.tif]

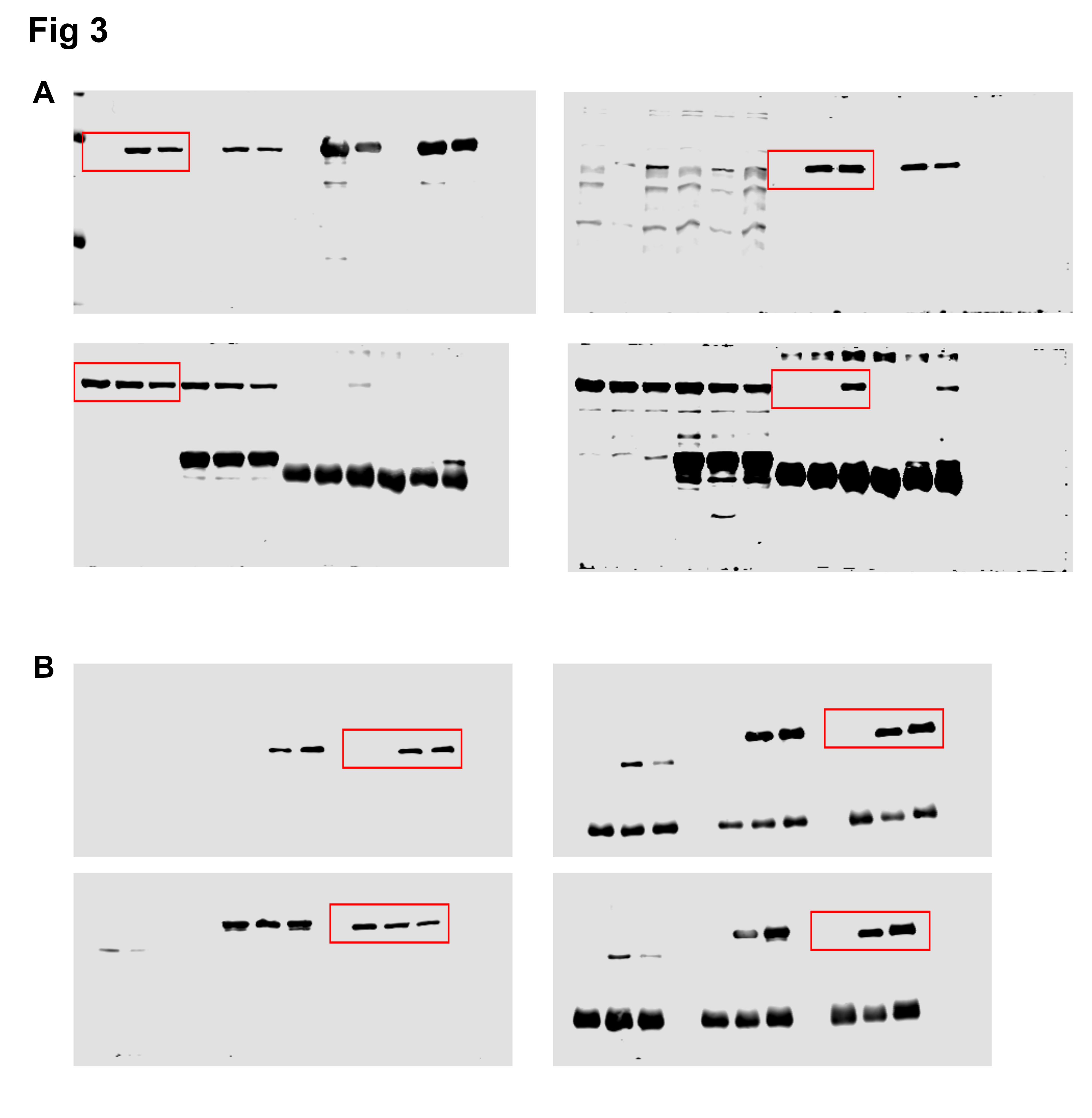

Supplement: Supplementary file 2 [file Data_Sheet_2.ZIP › Original WB images for 866057-2/Figure 3 original.tif]

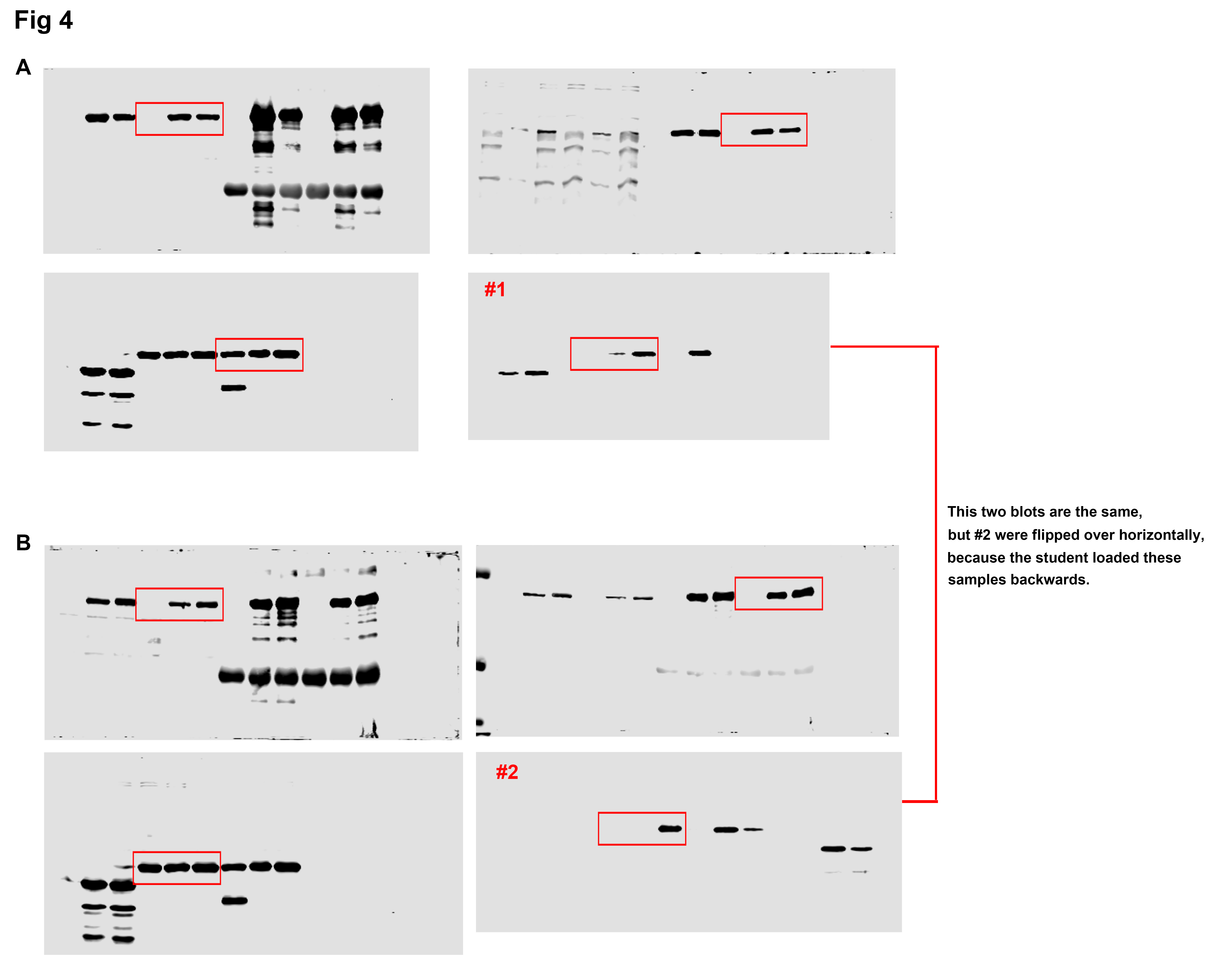

Supplement: Supplementary file 2 [file Data_Sheet_2.ZIP › Original WB images for 866057-2/Figure 4 original.tif]

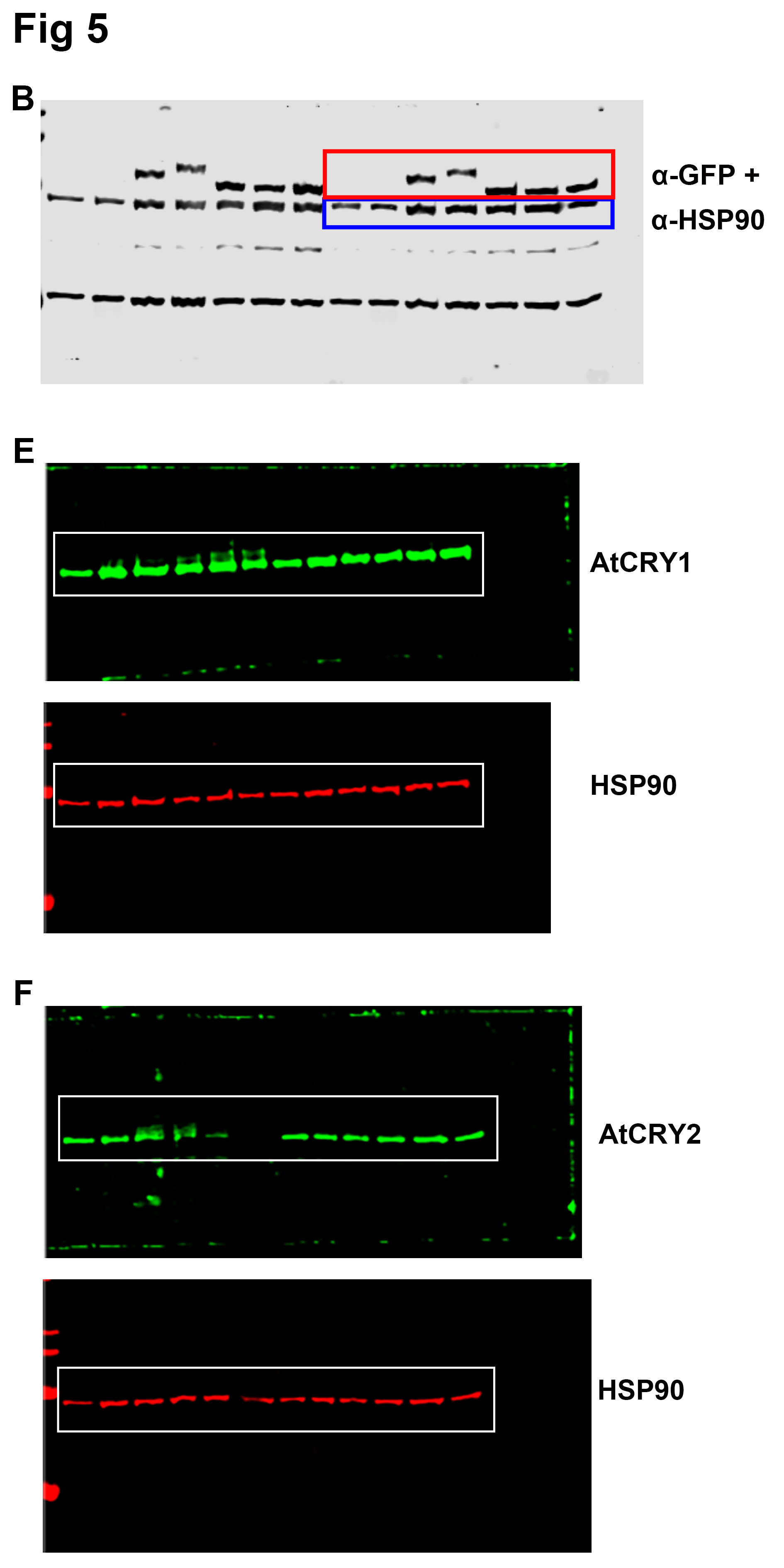

Supplement: Supplementary file 2 [file Data_Sheet_2.ZIP › Original WB images for 866057-2/Figure 5 original.tif]

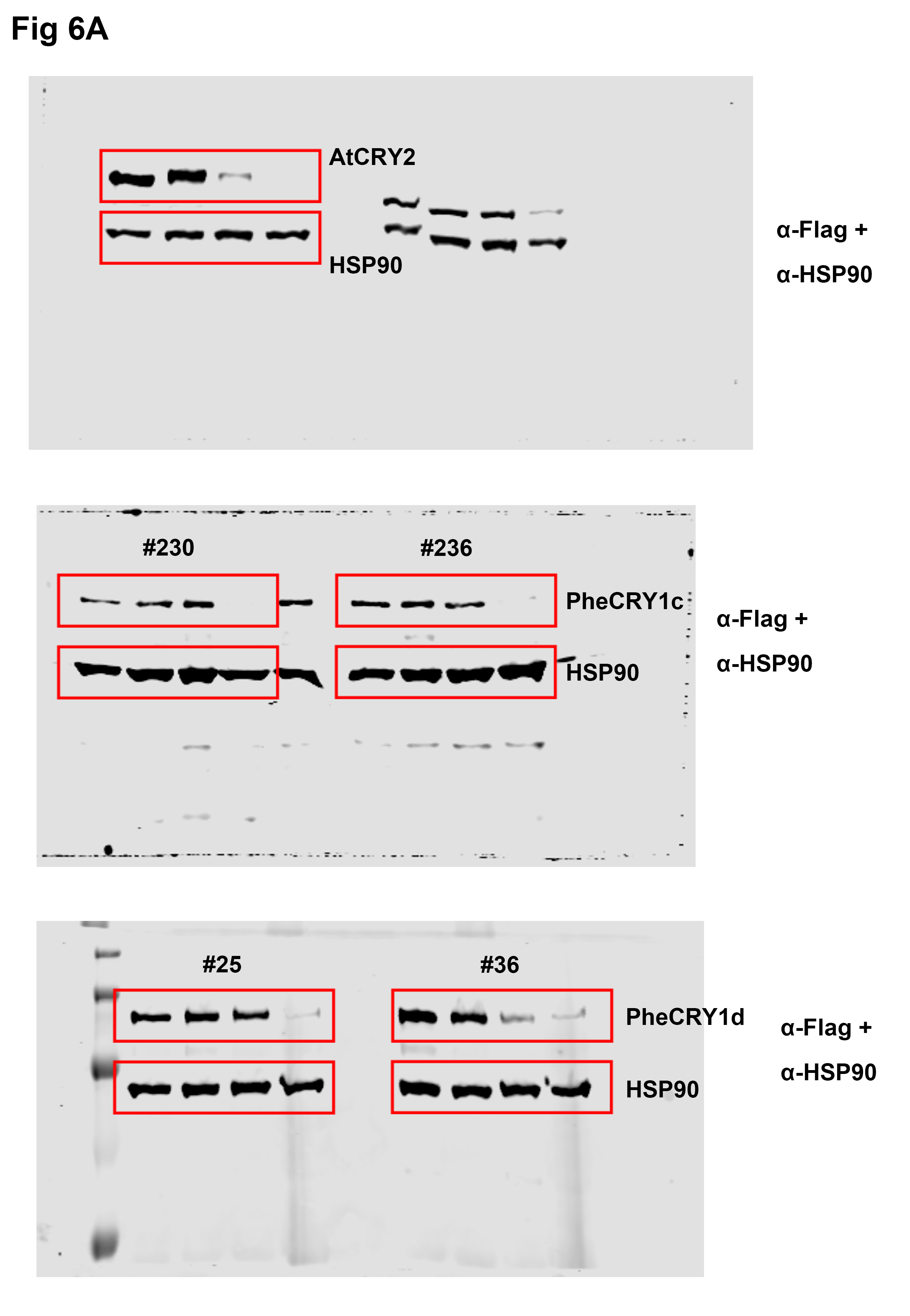

Supplement: Supplementary file 2 [file Data_Sheet_2.ZIP › Original WB images for 866057-2/Figure 6 original.tif]

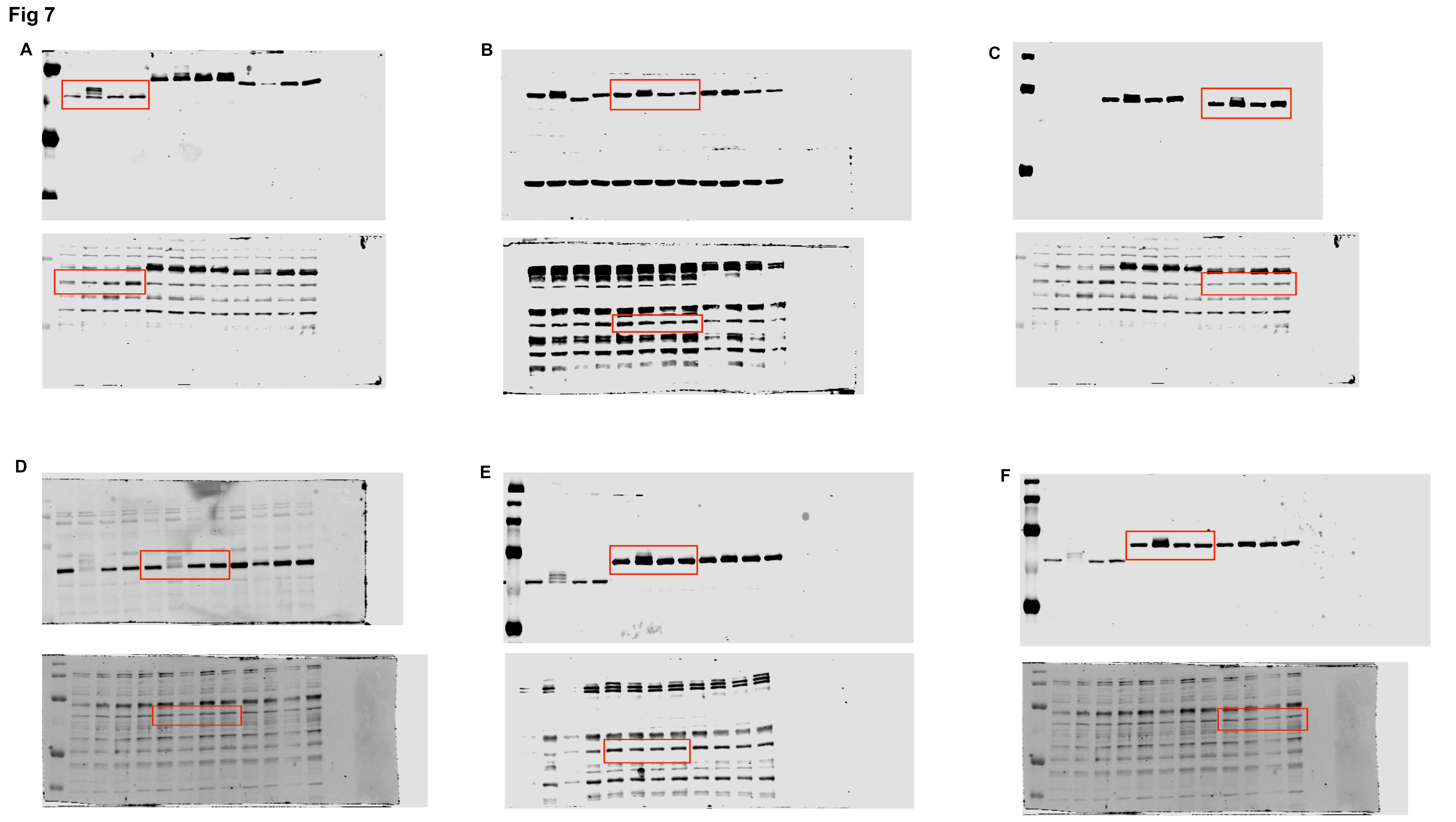

Supplement: Supplementary file 2 [file Data_Sheet_2.ZIP › Original WB images for 866057-2/Figure 7 original.tif]

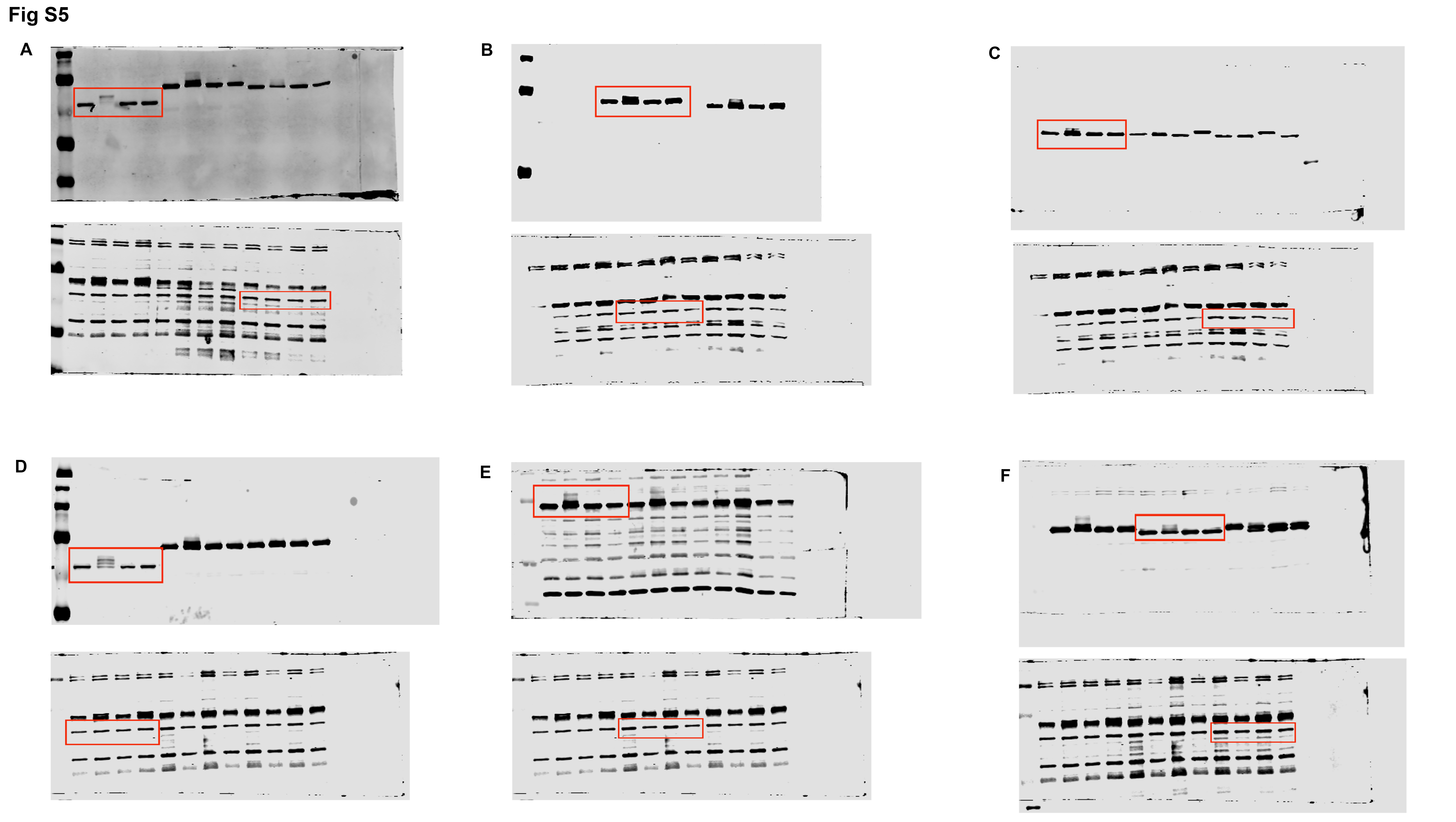

Supplement: Supplementary file 2 [file Data_Sheet_2.ZIP › Original WB images for 866057-2/Figure S5 original.tif]
